# Electronic Structure and Safety Insights into Prussian Blue Analog Cathode Behavior at Elevated Temperatures in Sodium-Ion Batteries

**DOI:** 10.1021/acs.energyfuels.5c03083

**Published:** 2025-09-22

**Authors:** Vadim Shipitsyn, Wenhua Zuo, Thanh-Nhan Tran, Tianyi Li, Sungsik Lee, Chanmonirath Michael Chak, Phung ML Le, Lin Ma

**Affiliations:** † Department of Mechanical Engineering and Engineering Science, 14727 The University of North Carolina at Charlotte, Charlotte, North Carolina 28223, United States; ‡ Battery Complexity, Autonomous Vehicle and Electrification (BATT CAVE) Research Center, The University of North Carolina at Charlotte, Charlotte, North Carolina 28223, United States; § Chemical Sciences and Engineering Division, 1291 Argonne National Laboratory, Lemont, Illinois 60439, United States; ∥ Energy and Environment Directorate, 6865 Pacific Northwest National Laboratory, Richland, Washington 99352, United States; ⊥ X-ray Science Division, 1291 Argonne National Laboratory, Lemont, Illinois 60439, United States; # Department of Applied Physical Sciences, 2331 University of North Carolina, Chapel Hill, North Carolina 27514, United States

## Abstract

Prussian blue analogs (PBAs) represent promising cathode materials for sodium-ion batteries (SIBs) due to their high theoretical capacity, open framework structure, and use of earth-abundant elements. However, the high-temperature structural evolution, water content effects, and thermal safety of PBAs, particularly in charged states, remain poorly understood, hindering their practical deployment. Here, we investigate Na_2_Fe­[Fe­(CN)_6_]·2H_2_O using thermogravimetric analysis (TGA), ex situ and in situ temperature-dependent X-ray absorption spectroscopy (XAS), and accelerated rate calorimetry (ARC). TGA and ex situ XAS confirm water loss between 150 and 200 °C, resulting in Fe^2+^ oxidation, enhanced local symmetry, and uniform redox behavior that improves electrochemical performance. In situ XAS reveals irreversible structural changes above 240 °C, including ligand loss, Fe site distortion, and increased disorder, while ARC on charged electrodes shows minimal self-heating rates (<0.1 °C/min) up to 300 °C, indicating exceptional thermal stability without lattice oxygen release. These insights elucidate PBA thermal dynamics, demonstrating improved electrochemical performance of water-deficient PBAs and informing future material design and safety assessment for SIB applications.

## Graphical abstract

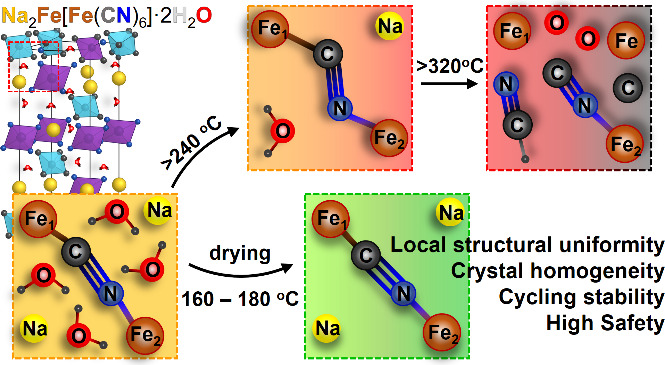

## 1 Introduction

Lithium-ion batteries (LIBs) have been pivotal in advancing global electrification through applications in grid energy storage and electric vehicles. However, escalating global energy demands necessitate alternative electrochemical storage solutions to reduce the reliance on scarce lithium and other critical metals. Sodium-ion batteries (SIBs) have emerged as a promising complement to LIBs, leveraging the abundant availability of sodium and electrode materials derived from earth-abundant elements such as carbon, manganese, and iron.^1^ SIB cathode materials primarily include layered oxides, polyanions, and Prussian blue analogs (PBAs).^2^ Among these, PBAs are attractive due to their high theoretical specific capacity, open framework structure, and straightforward synthesis.^3,4^ Notably, iron-based, vacancy-free, sodium-containing PBAs, such as Na_2–x_Fe­[Fe­(CN)_6_]·zH_2_O, offer a theoretical capacity of 171 mAh g^–1^ (for x, z = 0).^5^

The most common way to synthesize PBAs is aqueous precipitation routes,^6,7^ which lead to a high amount of interstitial and absorbed water in the open framework with a large interstitial space of PBAs. However, the presence of crystal water that incorporated during PBA synthesis can trigger detrimental side reactions, including electrolyte decomposition (e.g., forming HF via reaction with PF_6_^–^ anions^8^), electrode corrosion, and capacity fade. Heat treatment at elevated temperatures to remove water from pristine PBAs is critical for optimizing electrochemical performance.^9,10^ Recent reports^9–12^ investigated the importance of removing water from the PBA structure using techniques such as ex- and in situ X-ray diffraction (XRD), thermal gravimetric analysis (TGA), and ex-situ X-ray absorption spectroscopy (XAS) to explore the effect of structural changes on PBA electrochemical performance. For example, Wang et al. reported the crystal structure changes of Na_1.76_Fe­[Fe­(CN)_6_]·2.6H_2_O from trigonal to coexisiting phases in the range 150–300 °C and the final transformation to cubic phase after 300 °C leveraging high-temperature in situ XRD.^9^ They emphasized that the redox reaction of low Fe^2+^/Fe^3+^ is activated after heat treatment with improvement of the electrochemical performance. At the same time according to Xie et al. water molecules can be deintercalated from the structure as Na­(OH_2_)^+^ within the first cycle leading to low capacity and generation of HF.^13,14^ While prior studies have explored water removal’s impact on PBA electrochemical properties and phase transitions, the local structural evolution as a function of temperature remains underexplored. Similarly, the behavior of charged PBAs at elevated temperatures, critical for battery safety, has received limited attention. Despite PBAs’ oxygen-free structures and strong cyanide–transition-metal coordination conferring thermal stability,^15^ potential cyanide group evolution at high temperatures may pose safety risks through reactions with electrolytes.

To address these knowledge gaps, this study utilizes Na_2_Fe­[Fe­(CN)_6_]·2H_2_O as a model PBA cathode material, employing XAS and accelerated rate calorimetry (ARC) to probe the high-temperature behavior of pristine and charged states, respectively. XAS was used to monitor temperature-induced changes in the oxidation state and the local coordination environment of iron, providing insights into the structural transformations associated with water loss and thermal activation. Complementarily, ARC was applied to evaluate the thermal reactivity of the charged cathode in electrolyte-containing environments, assessing its propensity for exothermic reactions and thermal runaway. Together, these methods offer a comprehensive understanding of both structural stability and thermal safety, guiding the rational design of PBA cathodes with improved performance and safety profiles for next-generation SIBs.

## 2 Results and Discussion

Commercially available sodium-rich Na_2_Fe­[Fe­(CN)_6_]·2H_2_O was procured from a supplier, ensuring scalability for SIB applications. The long-range structure of pristine Na_2_Fe­[Fe­(CN)_6_]·2H_2_O powder was characterized via XRD, as shown in Figure 1A. The XRD patterns confirm a single-phase rhombohedral structure of R3 space group (lattice parameters: a = 7.4654 Å, c = 17.5887 Å, V = 848.94 Å^3^, Table S1), with sharp diffraction peaks indicating high crystallinity and minimal [Fe­(CN)_6_]^4–^ defects in the framework, consistent with prior reports.^11^ The split diffraction peaks of the (220) planes near 24° reflect reduced symmetry due to increased Na^+^ content per formula unit.^11,16^ SEM images (Figure 1B) reveal uniform microcubes (3–10 μm) of the Na_2_Fe­[Fe­(CN)_6_]·2H_2_O samples, confirming consistent morphology.

**Figure 1:**
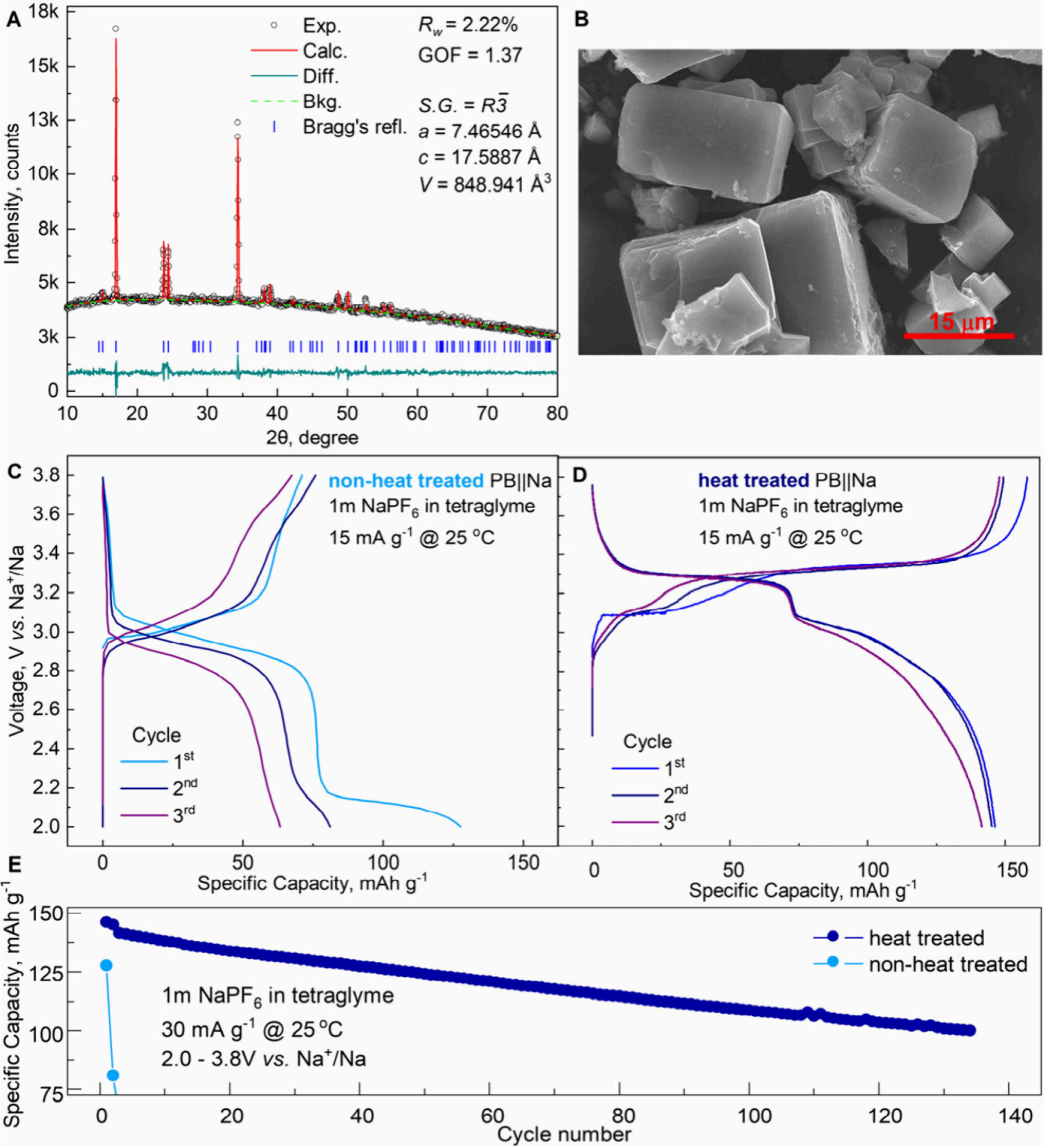
Characterizations of Na_2_Fe­[Fe­(CN)_6_]·2H_2_O material: (A) Rietveld refinement of X-ray diffraction pattern; (B) scanning electron microscopy of pristine material; cycle profiles for (C) nonheat-treated and (D) heat-treated (180 °C under vacuum for 48h) electrodes; (E) specific discharge capacity vs cycle number for Na_2_Fe­[Fe­(CN)_6_]·2H_2_O/Na half-cells cycled with 1m NaPF_6_ in tetraglyme at 25 °C.

Several studies have reported that absorbed and interstitial water in PBA cathodes for SIBs impairs electrochemical performance by reacting with electrolytes, triggering undesirable side reactions.^9,10^ Consequently, heat treatment to remove water is a critical processing step for PBA cathodes including the sodium-rich Na_2_Fe­[Fe­(CN)_6_]·2H_2_O material studied here. According to a previous report^9^ heat treatment of pristine Na_2_Fe­[Fe­(CN)_6_]·2H_2_O between 150 and 300 °C corresponds to a mixed trigonal-cubic coexisting phase, which can be easily rehydrated by air moisture due to the open framework of the material. To evaluate the impact of water content on the electrochemical performance of Na_2_Fe­[Fe­(CN)_6_]·2H_2_O material, galvanostatic charge–discharge curves were obtained for pristine and heat-treated Na_2_Fe­[Fe­(CN)_6_]·2H_2_O samples within a 2.0–3.8 V vs Na/Na^+^ potential window (Figure 1C–D). Both samples were cycled three times at 0.1 C (1 C = 150 mA g^–1^) at 25 °C. The heat-treated sample achieved a specific capacity of ∼ 145 mAh g^–1^ (Figure 1D), whereas the pristine sample exhibited a lower capacity of ∼ 125 mAh g^–1^ in the first cycle, followed by rapid capacity decay (Figure 1C). In Figure 1D, two plateaus are shown for the heat treated sample: 2.9–3.1 V corresponds to a high-spin (HS) Fe2–N Fe^2+^/Fe^3+^ reaction, and 3.3–3.5 V corresponds to a low-spin (LS) Fe1–C Fe^2+^/Fe^3+^ reaction.^16,17^ However, due to high amount of water in the structure, the nonheat treated sample does not have an activated LS Fe1–C Fe^2+^/Fe^3+^ redox reaction, which tremendously decreases the specific capacity of the electrode (Figure 1C). Nevertheless, we can clearly see another plateau at 2.2 V vs Na^+^/Na during discharge of nonheat treated sample (Figure 1C). Based on previous studies,^9,13^ this suggests extraction of water or Na­(OH_2_)^+^ during the initial cycles. Furthermore, the disappearance of this plateau after the second cycle indicates that water extraction occurs at ∼ 2.2 V vs Na^+^/Na. During long-term cycling at C/5 at 25 °C (Figure 1E), the heat-treated sample retained >70% capacity after 140 cycles, while the pristine sample showed significant capacity loss after three cycles, likely due to water reacting with the electrolyte, forming HF, and degrading cell performance.^8^

Differential capacity (dQ/dV) analysis was performed to evaluate the redox behavior of the Na_2_Fe­[Fe­(CN)_6_]·2H_2_O/Na half-cells during cycling. The PBA framework comprises LS Fe1–C and HS Fe2–N octahedra connected by Fe1–C≡N–Fe2 bonds.^9,16,17^ In dQ/dV profiles (Figure S1), the HS Fe2–N redox peak occurs at 2.9–3.1 V, and the LS Fe1–C peak is at 3.5–3.7 V. Over cycling, the HS Fe2–N peak in both pristine and heat-treated samples exhibits significant attenuation and voltage shift, indicating increased resistance and slower ion kinetics.^16^ The LS Fe1–C peak remains stable in the heat-treated sample but gradually intensifies in the pristine sample, suggesting initial inactivity due to interstitial water in the nonheat treated sample.

Heat treatment is essential for removing water from PBA cathodes, significantly enhancing their electrochemical performance. To elucidate the influence of heat treatment on the electronic structure of iron ions during electrochemical cycling (Figure S2), normalized X-ray absorption near-edge structure (XANES) spectra at the Fe K-edge were analyzed (Figure 2A-B). The Fe K-edge reveals distinct effects of heat treatment on the oxidation state and the local structural uniformity of transition metals. Key XANES regions include pre-edge peaks (7111–7120 eV), attributed to 1s → 3d transitions, and the main peak (7126–7132 eV), corresponding to 1s → 4p transitions.^18,19^ In fresh cathodes, heat-treated Na_2_Fe­[Fe­(CN)_6_]·2H_2_O exhibits predominantly Fe^3+^ with the main peak at 7132 eV, while nonheat treated Na_2_Fe­[Fe­(CN)_6_]·2H_2_O shows mixed Fe^2+^/Fe^3+^ states with the main peak at 7126 eV. The main peak shift in the heat-treated Na_2_Fe­[Fe­(CN)_6_]·2H_2_O sample results from water removal, which alters the iron oxidation state. Quantifying the removed water is challenging due to the high air sensitivity of PBA, which readily absorbs environmental moisture. Nevertheless, qualitative results of ex-situ XAS represent the successful drying process of Na_2_Fe­[Fe­(CN)_6_]·2H_2_O. Pre-edge peak intensity variations indicate ligand environment perturbations, with nonheat treated Na_2_Fe­[Fe­(CN)_6_]·2H_2_O displaying lower Fe oxidation and reduced centrosymmetry compared to heat-treated Na_2_Fe­[Fe­(CN)_6_]·2H_2_O.^18,19^ These features show that the extraction of H_2_O molecules from the structure leads to the oxidation of Fe to maintain the neutrality of the compound.^12,20^ Charged heat-treated Na_2_Fe­[Fe­(CN)_6_]·2H_2_O shows a main peak shift to ∼ 7127–7130 eV with splitting into two peaks, reflecting nonuniform redox behavior,^12,20–22^ which resolves after one cycle, indicating structural stabilization^20,21^ (Figure 2B). In contrast, charged pristine Na_2_Fe­[Fe­(CN)_6_]·2H_2_O exhibits persistent edge splitting and structural inhomogeneity, likely due to interstitial water-induced Fe^2+^/Fe^3+^ phase-separated domains^21^ (Figure 2A). At 2.0 V discharge, both samples display a dominant Fe^2+^ state, with enhanced pre-edge intensity (increased 3d–4p mixing) and a single main peak at 7126.5 eV, consistent with a symmetric FeN_6_ environment.^20–22^

**Figure 2:**
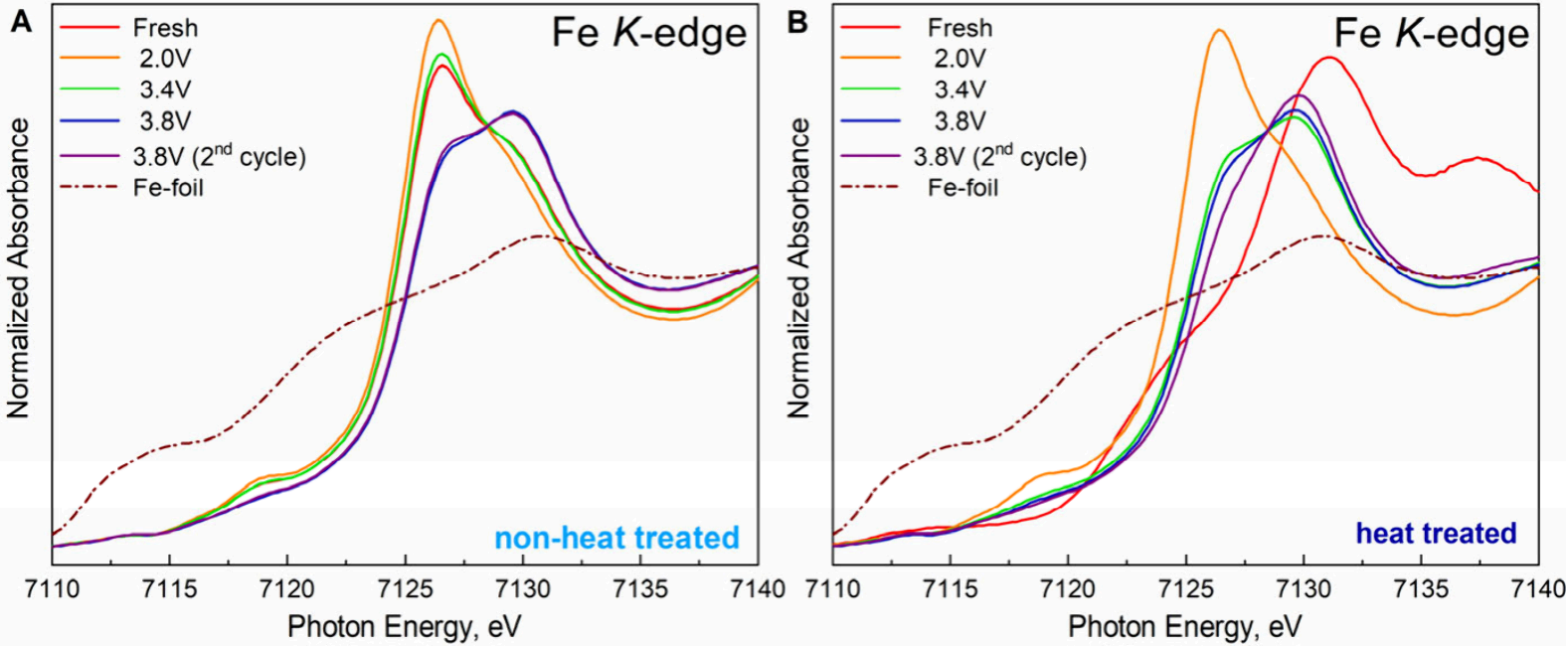
XANES of ex-situ XAS of Fe K-edge: (A) nonheat treated and (B) heat treated Na_2_Fe­[Fe­(CN)_6_]·2H_2_O material.

To investigate the temperature-dependent local structural changes in Na_2_Fe­[Fe­(CN)_6_]·2H_2_O cathodes, TGA coupled to temperature-dependent XAS was employed. TGA (Figure 3A), conducted under a nitrogen atmosphere, revealed three distinct weight-loss stages, consistent with previous reports.^23^ Pristine Na_2_Fe­[Fe­(CN)_6_]·2H_2_O exhibits a 9–10 wt % mass loss between 150 and 200 °C, attributed to the removal of adsorbed and interstitial water.^17^ Mass loss continues at a similar rate from 200 to 400 °C, accelerating above 400 °C, likely due to the decomposition of Fe–C≡N–Fe bonds, releasing CN^–^ groups as HCN and (CN)_2_ gas.^24^

**Figure 3:**
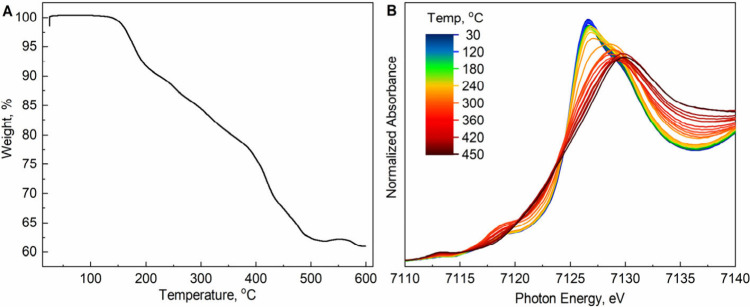
High-temperature experiments of pristine Na_2_Fe­[Fe­(CN)_6_]·2H_2_O: (A) TGA result; (B) in situ XANES of Fe K-edge as a function of temperature from 30 to 450 °C.

Temperature-dependent XAS of Na_2_Fe­[Fe­(CN)_6_]·2H_2_O reveals the thermal evolution of its local structure and Fe valence states (Figure 3B). From 30 to 180 °C, XANES spectra remain largely unchanged, with minor variations emerging at 200–220 °C. Irreversible structural changes occur above 240–260 °C, marked by significant XAS spectral shifts. At ∼ 7113–7114 eV, the pre-edge peak intensity increases, indicating 4p–3d orbital mixing and local Fe site distortion, likely reflecting Fe^2+^ coordination changes due to ligand loss along a centrosymmetric axis.^18–20,22,25^ At ∼ 7118–7119 eV, pre-edge peak intensity rises from 250 °C, peaking at 320 °C, suggesting Fe^2+^ oxidation to Fe^3+^ with octahedral distortion, possibly due to interstitial water removal.^19^ Above 320 °C, this intensity decreases, indicating an intermediate phase with altered Fe–C≡N bond lengths and potential Na^+^ or H_2_O displacement, followed by structural degradation and loss of Fe–C≡N–Fe bonds, potentially forming Fe oxides or carbides.^20–22,26^ At ∼ 7120–7124 eV, the loss of fine structure reflects increased disorder and a less symmetric local environment.^20^ At ∼ 7126–7132 eV, the main edge peak intensity decreases with temperature, suggesting fewer available Fe 4p states, indicative of increased disorder, while a main edge shift from ∼ 7126 to ∼ 7130 eV points to Fe^2+^ oxidation to Fe^3+^ or Fe–ligand bond shortening due to dehydration.^12,20–22,26^ Extended X-ray absorption fine structure (EXAFS) was Fourier transformed to analyze temperature-dependent changes in the crystal structure of Na_2_Fe­[Fe­(CN)_6_]·2H_2_O (Figure S3). The first shell peak (1.95–2.00 Å) corresponds to Fe1–C.^26,27^ Loss of Fe1–C≡N integrity begins after 220–240 °C and may indicate partial decomposition of cyanide ligands,^28^ increased thermal disorder, and reforming of Fe1–C≡N bonds. The second shell peak (2.35–2.40 Å), associated with the Fe2–N≡C–Fe1 bridge, exhibits disordering at 300–320 °C, likely due to water loss and partial CN^–^ dissociation.^12,26,28^ The peak at 4.50–4.70 Å, representing the Fe1···Fe2 distance via the cyanide bridge, decreases at 120 °C, indicating weakened long-range coherence from interstitial water removal.^21^ This peak stabilizes until 210 °C, possibly due to framework reorganization, but degrades significantly above 320 °C, reflecting framework collapse, Fe1–C≡N–Fe2 bridge breakdown, or formation of disordered phases (e.g., Fe oxides or carbides).^28^

In-situ XAS of Na_2_Fe­[Fe­(CN)_6_]·2H_2_O at increasing temperatures reveals thermally induced charge redistribution, local structural distortion, and increased disorder in the PBA framework, potentially accompanied by decomposition of CN^–^ into gaseous HCN or (CN)_2_. This raises concerns about CN^–^ reactions with electrolytes at elevated temperatures, which could influence self-heating rates (SHRs) and exacerbate thermal runaway risks, critical for SIB safety. To address these concerns, the thermal reactivity of charged Na_2_Fe­[Fe­(CN)_6_]·2H_2_O cathodes in the presence of an electrolyte was evaluated using ARC at elevated temperatures. Na_2_Fe­[Fe­(CN)_6_]·2H_2_O was preheat-treated to ensure optimal electrochemical performance and charged to 3.8 V vs Na/Na^+^ in 1 m NaPF_6_/tetraglyme electrolyte. To ensure consistent comparisons, the electrode material in each ARC tube was standardized to a capacity of ∼ 10 mAh. Figure 4 shows the SHR of the Na_2_Fe­[Fe­(CN)_6_]·2H_2_O cathode at 3.8 V vs Na/Na^+^ as a function of temperature. Notably, only minor exothermic activity was observed between 50 and 350 °C, with a maximum SHR of ∼0.1 °C/min. This decreased thermal response is likely attributable to the absence of entire lattice oxygen release, suggesting that Na_2_Fe­[Fe­(CN)_6_]·2H_2_O cathodes pose a low risk of thermal runaway, enhancing their safety for SIB applications. However, the potential formation of toxic HCN or (CN)_2_ during the thermal reaction process warrants careful consideration and requires a further detailed investigation.

**Figure 4:**
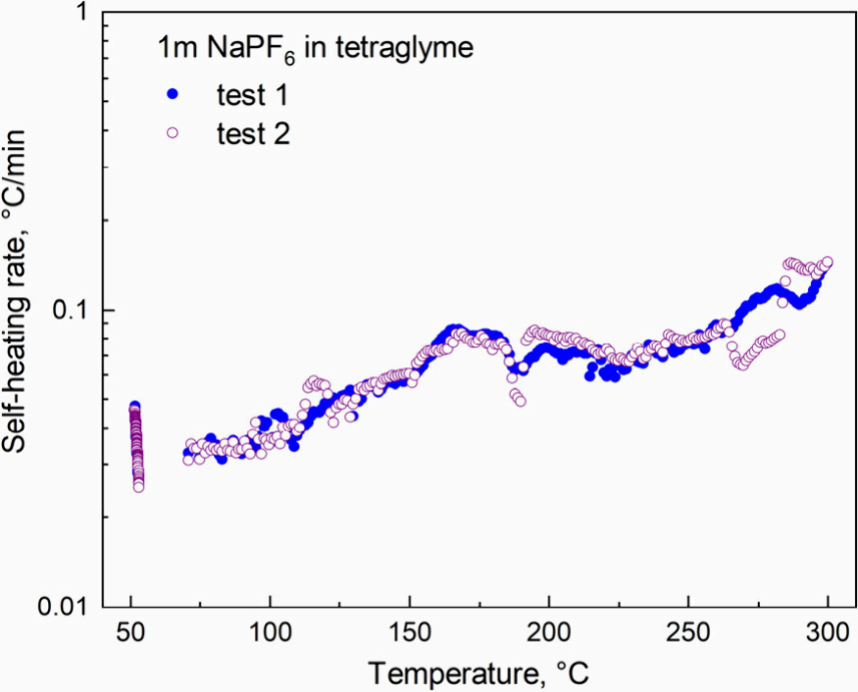
SHR vs temperature for reactions between charged Na_2_Fe­[Fe­(CN)_6_]·2H_2_O with electrolyte at elevated temperatures.

## 3 Conclusions

In this study, Na_2_Fe­[Fe­(CN)_6_]·2H_2_O was employed as a model PBA cathode material to investigate its high-temperature behavior from two perspectives. First, to elucidate the role of water removal in enhancing Na_2_Fe­[Fe­(CN)_6_]·2H_2_O electrochemical performance, ex-situ XAS results revealed that water removal leads to oxidation of Fe^2+^ to Fe^3+^, showing the enhancement of local structural symmetry, and more uniform redox behavior during cycling. TGA analysis showed the drop in weight (9–10 wt %) from 150 to 200 °C, resulting in the removal of absorbed and interstitial water. In-situ XAS was used to probe the local structure and Fe valence evolution as a function of temperature. Upon heating, temperature-dependent XAS showed progressive ligand loss, Fe site distortion, and increased disorder with irreversible structural decomposition above 240 °C, highlighting the electronic structural changes that underpin the enhanced stability and performance of the heat-treated cathode. Second, ARC tests demonstrated that charged Na_2_Fe­[Fe­(CN)_6_]·2H_2_O cathode exhibits minimal SHR, attributed to the absence of lattice oxygen release during electrolyte reactions at elevated temperatures up to 300 °C. These findings illustrate the importance the high-temperature behavior of Fe-based PBA cathode, demonstrating that low-water-content or water-free PBA materials enhance electrochemical performance and improve the safety of commercial SIBs by mitigating thermal runaway risks.

## Supplementary Material



## Notes

Further experimental details, extended X-ray absorption fine structure and electrochemical data (PDF)
