# Supplementary material for: Electronic Structure and Safety Insights into Prussian Blue Analog Cathode Behavior at Elevated Temperatures in Sodium-Ion Batteries
Source: Energy Fuels. 2025 Sep 22;39(39):19054–9. doi: 10.1021/acs.energyfuels.5c03083 (PMC12498420; doi:10.1021/acs.energyfuels.5c03083)
Supplement: Supplementary file 1 [file ef5c03083_si_001.pdf]

Supporting Information

Electronic Structure and Safety Insights into Prussian Blue Analog Cathode Behavior at Elevated Temperatures in Sodium-Ion Batteries

Vadim Shipitsyn[a,b], Wenhua Zuo[c], Thanh-Nhan Tran[d], Tianyi Li[e], Sungsik Lee[e], Chanmonirath (Michael) Chak[a,b], Phung ML Le[d], Lin Ma[a,b,f]*

a. Department of Mechanical Engineering and Engineering Science, The University of North Carolina at Charlotte, Charlotte, NC 28223, USA
b. Battery Complexity, Autonomous Vehicle and Electrification (BATT CAVE) Research Center, The University of North Carolina at Charlotte, Charlotte, NC 28223, USA
c. Chemical Sciences and Engineering Division, Argonne National Laboratory, 9700 S Cass Ave, Lemont, IL 60439 USA
d. Energy and Environment Directorate, Pacific Northwest National Laboratory, Richland, WA 99352 USA
e. X-ray Sciences Division, Argonne National Laboratory, Lemont, IL 60439, USA
f. Department of Applied Physical Sciences, University of North Carolina, Chapel Hill, NC 27514, USA

E-mail: l.ma@unc.edu

Experimental Details

1. Materials preparation:

$Na_2Fe[Fe(CN)_6]\cdot 2H_2O$ was provided by Altris (Sweden). To remove moisture and optimize electrochemical performance for cycling tests, $Na_2Fe[Fe(CN)_6]\cdot 2H_2O$ material was heat-treated under vacuum at 180 °C for 48 hours. Sodium hexafluorophosphate ($NaPF_6$, > 99%) salt was acquired from CapChem Technology, while tetraethylene glycol dimethyl ether (TEGDME) was sourced from TCI Chemicals. The solvent was treated with a molecular sieve until the moisture content was reduced to below 20 ppm prior to its use in electrolyte preparation.

2. Materials characterization:

Lab-source powder X-ray diffraction (XRD) analysis was conducted using a Rigaku Miniflex 600 diffractometer equipped with Cu Kα radiation (wavelength of 1.5406 Å). Scanning electron microscopy (SEM) was used to investigate morphology of material using JEOL JSM 6480 SEM with accelerating voltage 30 kV.

Thermogravimetric analysis (TGA) was tracked for pristine material in a platinum crucible from room temperature to 600 ^{o}C with ramp 1 ^{o}C/min in nitrogen environment using Discovery TGA 5500 analyzer.

Ex situ X-ray absorption spectroscopy (XAS) for the Fe (7112 eV) K-edge was collected at the 7-BM (QAS) beamline of the National Synchrotron Light Source-II (Brookhaven National Lab (BNL), USA) in transmission mode. Fe metal foil was used as the standard references to calibrate energy shifts. $FeSO_4\cdot 7H_2O$ (98% Thermo Fischer Scientific), Fe_2O_3 (99.9% Thermo Fischer Scientific) were used as references of the transition metal oxidation states. These references were prepared by mixing sucrose (C12H22O11) with the chemicals contained transition metal in the needed stable oxidation state to achieve the mixture with 5%wt. of the studied metal. Mixed chemicals were pelletized (0.1g) and sealed in Kapton film to prevent influence of air environment. Electrodes (active material, binder, carbon black in the 90:5:5 wt.% ratio) on the Al foil (⌀ 11mm with ~6.0 mg cm^{-2} average active material loading) of pristine and heat treated samples were prepared in different states of charge. Five cells for each electrode material charged to 3.4V, 3.8V after one full cycle) and discharged to 2.0 V (after charging to 3.8V) vs. Na+/Na in CC(15 mA g^{-1}) – CV(1.5 mA g-1) mode were recovered from the two-electrode cells (with Na metal) in Ar-filled glovebox and sealed in Kapton film. Four spectra were collected for each K-edge and merged to improve signal-to-noise ratio.

In situ heating XAS for the Fe (7112 eV) K-edge was collected in transmission mode at 12-BM at Advanced Photon Source (APS). The 12BM beamline at the APS at the Argonne National Laboratory is equipped with a Si(111) monochromator. XAS data of metal foils and other reference materials were also collected in the transmission mode for X-ray energy calibration and data alignment. XAS spectra were registered in-situ from 30 to 450°C for pristine $Na_2Fe[Fe(CN)_6]\cdot 2H_2O$ powder.

Athena software program package was used to analyze X-ray absorption near edge structure (XANES) spectra. Background, pre-edge and post-edge lines were defined to normalize XANES spectra. The extended X-ray absorption fine structure (EXAFS) spectra were Fourier transformed in the 3.0 – 13.7 Å^{-1} k-range.

Accelerating rate calorimetry (ARC) analysis was performed using a MMC 274 Nexus calorimeter (NETZSCH). Pristine $Na_2Fe[Fe(CN)_6]\cdot 2H_2O$ powder was dried at 180 °C within 48 hours, transferred to glovebox and mixed with acetylene black conductive agent (Timcal C45) and polyvinylidene (PVDF, Solef 5130) in a weight ratio of 90:5:5 wt.%. Prepared mixture was dissolved in N-methyl pyrrolidone (NMP), spread in a mortar and dried in vacuum antechamber within several hours. Dried powder (~200mg) was pelletized without air exposure, and the pellet was used as a cathode in half-cell with metal sodium. Pellet was charged to 3.8V vs. Na^+/Na in two-electrode cell configuration vs. Na metal with dried glass-fiber (Whatman, GF/A) separators using several steps of decreasing current (C/80, C/160, C/320, C/640, where 1C = 150 mA g^{-1}) with 15 minutes rest after each charge. Charged pellet was extracted, crushed, and 75 mg of the powder were sealed in stainless steel tube (⌀5mm, l = 40mm) with 25mg of 1m $NaPF_6$ (>99%, CapChem Technology) in tetraglyme (TEGDME, H_2O < 20 ppm, TCI Chemicals) electrolyte using inert gas welding machine inside Ar-filled glovebox. The ARC response was tracked from 50 to 300 ℃ temperature range under adiabatic conditions, when the self-heating rate (SHR) exceeded 0.03 ℃ min^{-1}.

3. Electrochemical measurements:

$Na_2Fe[Fe(CN)_6]\cdot 2H_2O$ electrodes were prepared for electrochemical testing by mixing active material with acetylene black conductive agent (Timcal C45) and polyvinylidene (PVDF, Solef 5130) in a weight ratio of 90:5:5. The mixture was dissolved in NMP to form viscous slurry. The obtained slurry was spread onto Al foil using a Dr. Blade and then dried in glove-box antechamber under vacuum overnight. The punched electrodes (⌀ 11mm with ~6.0 mg cm^{-2}) were dried at 110 ℃ under vacuum overnight. CR-2032 coin cells coupled with Na metal anode were tested using 1m $NaPF_6$ (>99%, CapChem Technology) in TEGDME (H_2O < 20 ppm, TCI Chemicals) electrolyte with polyethylene (PE, Celgard) separator in the voltage range of 2.0 – 3.8 V vs. Na^+/Na.

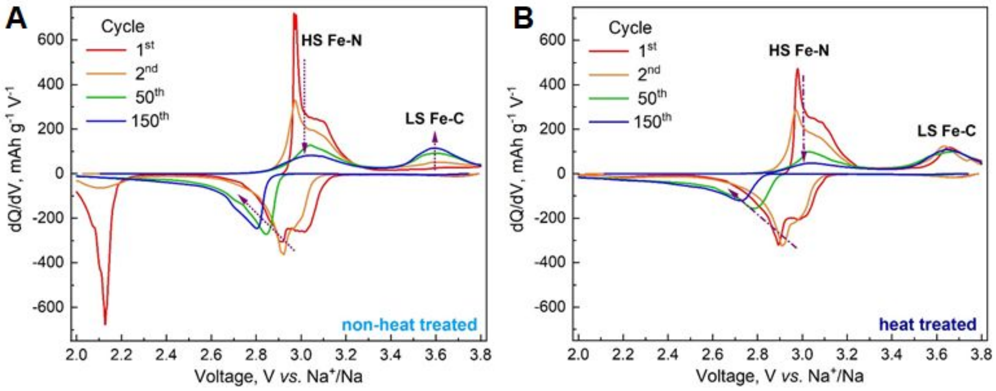

Figure S1. Differential analysis dQ/dV of $Na_2Fe[Fe(CN)_6] \cdot 2H_2O$ in half-cells for (a) non-heat treated and (b) heat treated samples.

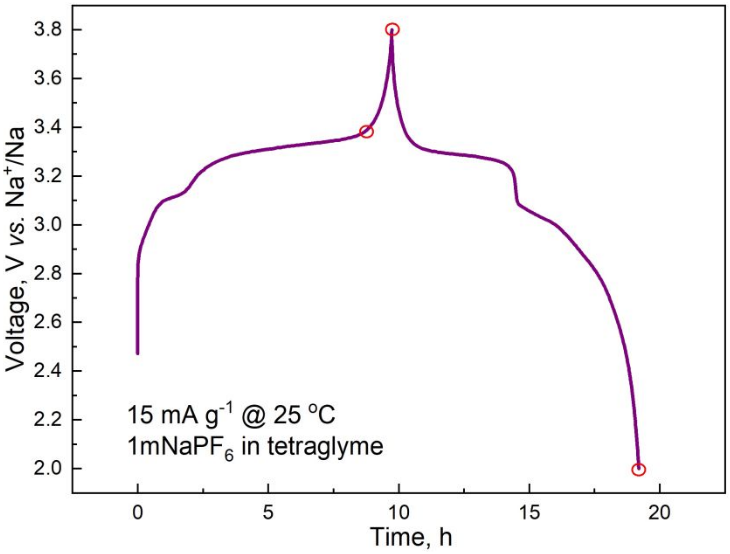

Figure S2. Voltage vs. time of $Na_2Fe[Fe(CN)_6]\cdot 2H_2O$/Na cells for ex-situ X-ray absorption spectroscopy experiment.

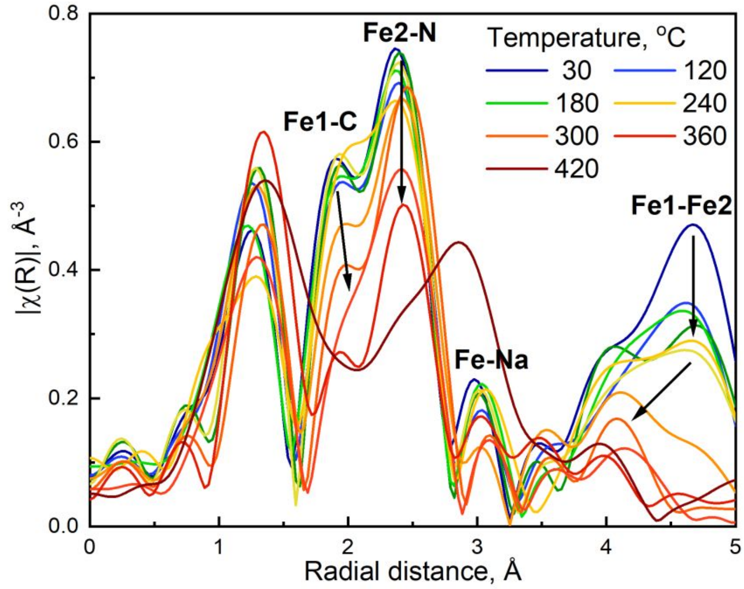

Figure S3. In-situ EXAFS of pristine $Na_2Fe[Fe(CN)_6] \cdot 2H_2O$ at elevated temperatures.

Table S1. A summary of XRD refinement parameters of pristine $Na_2Fe[Fe(CN)_6] \cdot 2H_2O$.

$Na_2Fe[Fe(CN)_6] \cdot 2H_2O$						
Space group: $R\overline{3}$						
a = 7.4656(8) Å; c = 17.5877(18) Å; V = 848.92(5) Å^3						
R_w = 2.22%; GOF = 1.37						
Atom	Site	x/a	y/b	z/c	Occupancy	U_{iso} (Å^2)
Na	6c	0.0000	0.0000	0.2391	0.8620	0.0080
Fe1	3a	0.0000	0.0000	0.0000	1.0000	0.0229
Fe2	3b	0.3333	0.6667	0.1667	1.0000	0.0020
C	18f	0.1511	0.2890	0.0796	1.0000	0.0130
N	18f	0.1991	0.3995	0.1019	1.0000	0.0218
